# Development and Psychometric Validation of a Usability Instrument Based on ISO 25010 for Electronic Health Record Systems in Peruvian Health Care Settings: Methodological Study

**DOI:** 10.2196/81377

**Published:** 2026-05-22

**Authors:** David Mamani-Pari, Alex Froilan Peñaloza-Hualpa, Milton Edward Humpiri-Flores, Eder Gutierrez-Quispe, Jorge Eddy Otazu-Luque

**Affiliations:** 1 Escuela Profesional de Ingeniería de Sistemas, Facultad de Ingeniería y Arquitectura, Universidad Peruana Unión, Carretera Arequipa Km-6, Juliaca, 21101, Peru, 51 951782520

**Keywords:** usability scale, human factors, electronic health record, ISO 25010, Peruvian health system, usability evaluation

## Abstract

**Background:**

The usability of electronic health records influences patient safety, clinical efficiency, and the sustainable digital transformation of health care. While tools such as the System Usability Scale exist, few are based on structured software quality models or align with local regulatory frameworks, particularly in low- and middle-income countries.

**Objective:**

This study aims to develop and psychometrically validate an instrument to assess electronic health record usability, based on the ISO 25010 (International Organization for Standardization) quality model, and in accordance with the Peruvian Technical Health Standard (Norma Técnica de Salud [NTS]).

**Methods:**

A 3-stage methodological study was conducted following COSMIN (Consensus-based Standards for the Selection of Health Measurement Instruments) guidelines. First, a 23-item instrument was developed, grouped into 7 usability dimensions based on ISO 25010. Second, content validity was assessed using a modified Delphi method with 10 experts, using Aiken’s V coefficient. Third, structural validity and reliability were evaluated in 115 participants (physicians, nurses, and administrative staff) using confirmatory factor analysis and Cronbach α. Additionally, the module’s functional compliance with the NTS was verified.

**Results:**

The instrument demonstrated excellent psychometric properties among 115 participants (53 physicians, 48 nurses, and 14 administrative staff); high content validity (Aiken’s V=0.95; 95% CI 0.84‐0.98), excellent internal consistency (*α*=.968), and optimal factor fit (Comparative Fit Index=1.000; Tucker-Lewis Index=1.001). Overall usability was perceived as very high by 94 of 115 users (81.7%), and high by 21 of 115 users (18.3%), with no perceptions at lower levels. The module met 28 of 35 requirements (80%) of the Peruvian Technical Health Standard. Administrative staff reported higher satisfaction (13/14, 93% rated it as very high) compared with clinical professionals (81/101, 80% rated it as very high), particularly in the dimensions of error protection and operability.

**Conclusions:**

This study provides the first fully psychometrically validated instrument in Spanish based on ISO 25010, System Usability Scale, and other theoretical foundations for evaluating the usability of information systems. The results highlight the importance of considering both international standards and local regulatory requirements (NTS) in the design of digital health systems. The instrument is applicable in Spanish-speaking contexts and can serve as a reference for future usability evaluations in the region as well as in low- and middle-income countries.

## Introduction

### Digitization and Usability in Electronic Health Record Systems

Electronic health record (EHR) systems have become essential tools for improving the quality of medical care, reducing errors, and optimizing clinical processes [1]. However, their effectiveness depends critically on their usability, particularly in high-workload health care settings such as Peru, where health care professionals face time and resource constraints [2]. From the patient’s perspective, studies have also been conducted on EHRs, highlighting the physician’s central role within the workflow. Evidence shows that physicians who use EHRs to exchange information with their patients, develop care plans, and provide other services tend to report higher satisfaction with computerized systems [3]. On the other hand, there are also studies revealing that the adoption of health information systems has led to medical errors attributed to technological failures, although there are certain perceptual discrepancies between clinical and information technology (IT) professionals regarding their causes. While IT experts attribute errors to technical failures such as poor interfaces and lack of training, physicians point to contextual and human factors such as workload, familiarity with new systems, and inadequate processes [4]; it is important to note that physicians work under very tight schedules and require IT tools that truly provide support; therefore, there are various challenges to be addressed in different parts of the world depending on culture, technological capacity, socioeconomic status, government policies, and other factors; thus, it remains a subject of study for various researchers [5-9].

### Context of the Peruvian Health Care System

Similarly, Peru faces challenges; given that the existence of fragmented medical records or the inability to locate a medical record—even within the same Healthcare Service Provider Institution (Instituciones Prestadoras de Servicios de Salud [IPRESS])—creates problems of information fragmentation and wasted man-hours in human resources due to the need to repeatedly fill out a new medical record, which hinders the provision of comprehensive, quality health care due to the lack of important information from previous medical records; this same issue is mentioned in the Peruvian government’s Technical Implementation Document for the National Registry of Electronic Medical Records (Registro Nacional de Historias Clínicas Electrónicas [RENHICE]) in section 6.3, which states the following: “In our country, almost all medical records are handwritten. If a patient visits an IPRESS for the first time, a new medical record is opened, which involves recording data (administrative and clinical) on forms that are part of the medical record. On the other hand, when the patient returns for a subsequent visit, the staff cannot locate their manual medical record (Historia Clínica Manual [HCM]); therefore, they must re-enter the data. Likewise, the procedure is repeated when a patient changes their address, changes IPRESS facilities, or is located anywhere else in the country. Currently, a patient has as many medical records as the number of IPRESS facilities they have visited, and they may have more than one record at the same IPRESS facility. In response to this problem, the government has established the Technical Health Standard (Norma Técnica de Salud [NTS]) for Medical Record Management; however, this cannot be resolved without an adequate computer system to assist in managing medical records within a specific IPRESS—a system that can be adapted to the institution’s specific needs while adhering to the Peruvian government’s NTS, given that it applies to all IPRESSes, whether public, private, or mixed.

### Regulatory Framework: NTS and the Need for Regulatory Alignment

Therefore, there is a need for a Clinical Record Management Information System that incorporates the criteria or considerations of the National Technical Standard (NTS) for Clinical Record Management; this system must meet usability criteria so that users are satisfied when using this tool. According to [10], the ease of use of a system can reduce errors, thereby contributing to efficiency and allowing physicians to spend more time with patients; hence the importance of evaluating usability, since studies cited in the research indicate that physicians experience usability issues, particularly when working with EHRs, which result in lengthy training periods and lost productivity. It is worth noting that usability could mean the difference between life and death; furthermore, it is argued that usability issues arise in medical devices, procedures, and even diagnostic tools; therefore, there is a need to conduct a study on the evaluation of the impact of NTS on the implementation of the Clinical Records Module in relation to its usability.

### Usability Gaps and Human Factors in EHRs

According to studies, what is now known as electronic medical records (EMR) has been in use since the 1980s, primarily in developed countries. Subsequent studies were conducted on their use, as it was perceived that physicians were resistant to change; consequently, it was observed that physicians adopt the use of these systems once they perceive their utility for their patients; thus, it was concluded that the key lies in ensuring that the software tools are easy to use and highly beneficial for the medical team [11]. The evaluation of EMR management tools is almost continuous, as it positively or negatively affects the care provided to patients by physicians. Therefore, they report having surveyed users—primarily physicians and nurses who interact directly with the tool in question—and found that while the tool facilitates greater data capture, it also impacts medical staff time, as they spend more time at the computer, which in some ways reduces the time available for patient care; consequently, they concluded that the tool has minimal impact on patient care. On the other hand, they showed minimal satisfaction with the tool; however, there was resistance to returning to handwritten records [12]. Another study also notes a minimal impact from the implementation of EMR systems, and the implementation of EMR management software in health care facilities is increasingly widespread [13].

Cultural diversity, economic conditions, and other factors influence the adoption of technological alternatives; therefore, it is recommended to have a plan that coordinates the entire EMR implementation process, considering the current organizational context, given that some countries face challenges, particularly those that are underdeveloped or developing [14]. This implies restructuring the curricula for training health care professionals, as well as providing EMR training to all staff at the institution [15]. On the other hand, studies are also being conducted on the impact of the EHR on patients by allowing them access to their records, thereby enhancing safety in medication management and providing greater control over medication [16].

The need for EHR management platforms in the health care systems of various countries is critical, as, despite the challenges, they are cost-effective to maintain because they enable faster searches, reducing the time and cost of locating patient records; consequently, they are being widely adopted worldwide [17]. These platforms can be classified into several types, such as government, commercial, organizational, or multi-organizational; however, it is important to highlight certain key characteristics they must meet, such as data interoperability, displaying the appropriate level of detail in information, and compliance with health care privacy regulations; the aforementioned aspects still pose a challenge in terms of compliance [18].

The transition to the EHR has been a fundamental advancement in the medical field, as it allows for the consolidation of clinical information, improves evidence-based decision-making, enhances access to information, and facilitates multidisciplinary collaboration. However, the implementation of EHRs has revealed significant challenges regarding efficiency, reliability, and the potential increase in medical-legal risk; that is, it is not a perfect solution; as medical malpractice lawsuits involving EHRs highlight documentation errors caused by various factors such as information fragmentation, access failures during system outages, issues with electronic data transfer, ineffective alerts, and a lack of integration among hospital systems (interoperability); which can compromise patient care and expose providers to legal liability. Therefore, the correct completion of documentation in the EHR is of utmost importance; errors in data entry generally occur due to the use of prefilled templates or the copying of unverified data; this necessitates continuous improvement of EHR systems to optimize their functionality, as well as the establishment of protocols to ensure that records are complete, accurate, and faithfully reflect the clinical assessment. In this way, it ensures that EHR platforms are not merely tools for storing information, but rather a comprehensive system supporting medical care that considers medical-legal aspects and patient safety [19]; hence the importance of EHR systems adhering to health sector regulations. It is also worth noting that there is a study evaluating the suitability of EHR data using a framework called ISPOR (International Society for Pharmacoeconomics and Outcomes Research) to ensure the relevance and validity of the information generated by EHRs; this framework includes 2 components: data delineation and data suitability for the purpose; each component involves a series of checklists [20]. Apart from the challenges mentioned above, interoperability is a critical factor for the success of health information systems, as it enables the secure and efficient exchange of patient data among different health care providers; however, there are many challenges with the integration and the automatic and secure exchange of information among the various EHR health care systems [21]. Consequently, organizations often face challenges in achieving successful technology implementation; therefore, it is of vital importance to conduct a comprehensive analysis of the opinions of internal and external users regarding EHR software decisions; this can help improve and optimize EHR information systems and reduce technology acceptance gaps [22].

The objective of this research was to design and psychometrically validate a usability evaluation instrument based on the ISO/IEC 25010 (International Organization for Standardization/International Electrotechnical Commission) standard for EHR systems in the Peruvian context. Specifically, the study sought to: (1) integrate the usability dimensions of ISO 25010 with the System Usability Scale (SUS) and other relevant theoretical foundations, constructing a comprehensive instrument in Spanish; (2) validate the instrument through expert judgment using Aiken’s V coefficient and confirm its psychometric properties through confirmatory factor analysis (CFA) and reliability analysis; and (3) evaluate the usability of an EHR module implemented in accordance with the Peruvian NTS, identifying the degree of regulatory compliance and differences in the perception of usability among different types of health care professionals (physicians, nurses, and administrative staff). This study aims to fill the gap identified in the literature regarding the lack of standardized instruments in Spanish, with psychometric validations that integrate international quality standards with local health sector regulations, providing a validated tool that can be replicated in similar contexts in Latin America, such as in low- and middle-income countries.

## Methods

### Study Design

**Figure 1.**
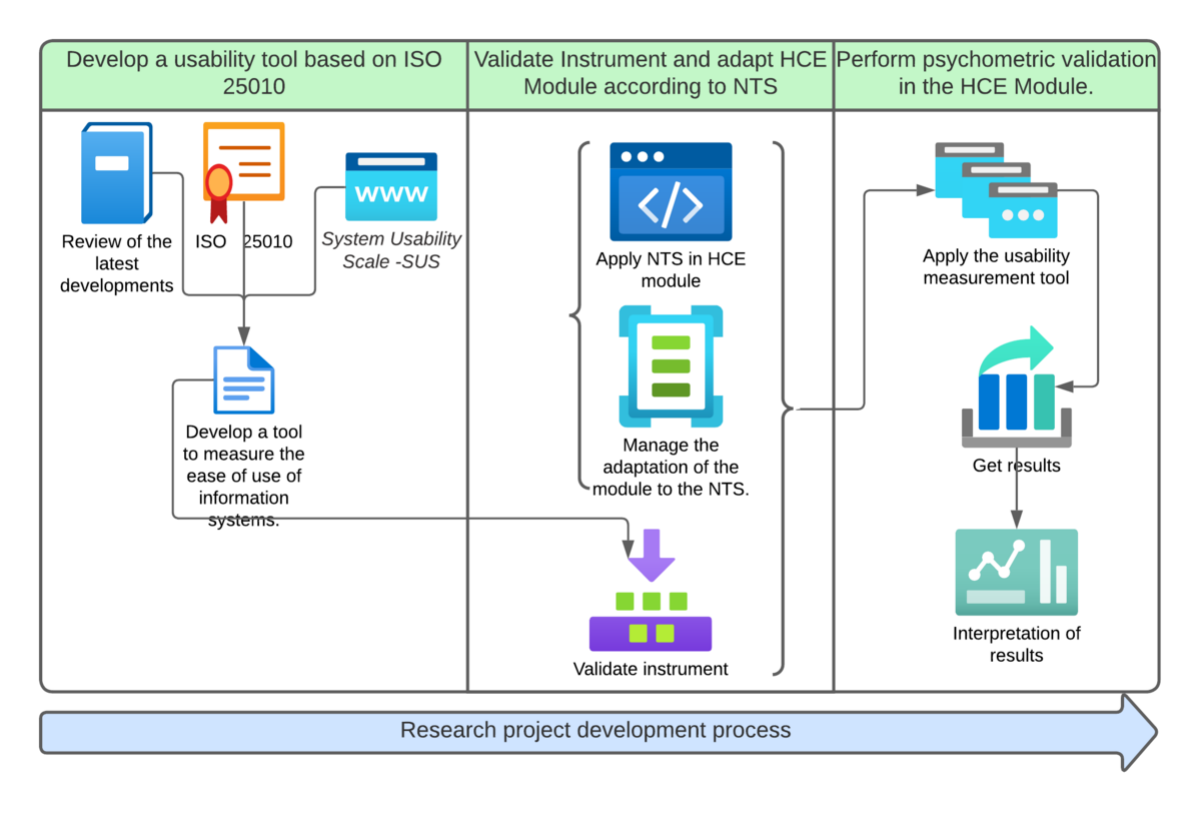
Research development methodology. HCE: Electronic Health Record (Historia Clínica Electrónica); ISO: International Organization for Standardization; NTS: Norma Técnica de Salud (Technical Health Standard); SUS: System Usability Scale.

This study followed a 3-stage sequential instrument development and validation methodology, aligned with the COSMIN (Consensus-based Standards for the Selection of Health Measurement Instruments) guidelines for psychometric studies in health. The first stage (theoretical development) integrated the usability attributes of ISO 25010, SUS, and other theoretical foundations on usability. The second stage (content validation) used the modified Delphi method with 10 experts, in addition to aligning the functionality of the electronic health record (Historia Clínica Electrónica [HCE]) module with the functional requirements of the Peruvian Technical Health Standard (NTS), while the third stage (structural validation and reliability) included the application of the instrument to 115 end users; this process is shown in Figure 1.

### Participation and Context

For content validation, a total of 10 experts were deliberately recruited, distributed as follows: 5 specialists in areas related to software development, 3 researchers with experience in systems evaluation, and 2 professionals from the health care sector. This selection aimed to incorporate the usability quality attribute, in accordance with the guidelines established in the ISO/IEC 25010 standard and the SUS questionnaire. This methodological approach has been previously used in similar studies [23,24].

For structural validation and reliability (psychometric), the study obtained an institutional authorization letter and involved 115 workers from a hospital network in the Puno region, distributed across the following professional categories: physicians (53/115, 46%), nurses (48/115, 42%), and administrative staff (14/115, 12%), all with at least 6 months of experience using the EHR module. The sample size was determined in accordance with the recommendation of 5 participants per item of the instrument (23 items → n=115), meeting the methodological criteria required for the application of CFA.

Likewise, it should be noted that ISO/IEC 25010:2011, which replaces the previous ISO/IEC 9126-1:2001, defines 2 models: a quality-in-use model and a product quality model, each with its corresponding characteristics. Regarding the in-use quality model, the aim is to quantify the “usability” of the application strictly related to the end user of the product [25].

### Development of the Instrument

The initial questionnaire consisted of 23 items organized into 7 dimensions, which were based on the quality model of the ISO/IEC 25010 standard: (1) functional adequacy, (2) learnability, (3) operability, (4) protection against user errors, (5) esthetic presentation, (6) accessibility [26], and (7) user satisfaction. Theoretical support regarding instruments and techniques for measuring “usability” was sought, and the decision was made to develop a Spanish-language instrument based on ISO/IEC 25010:2011 and supported by the SUS and other instruments addressing the subject in question. Subsequently, the modified Delphi technique [27,28] was followed, since the traditional technique was not strictly adhered to; however, given the nature of an instrument already theoretically grounded, supported by a review of the state of the art, at least 2 rounds were necessary to obtain adequate feedback from 10 expert judges who were selected as described in the previous subsection.

### Content Validity

The experts evaluated each item based on 3 criteria (relevance, appropriateness, and clarity) using a 5-point Likert scale (where 1 represents the minimum value and 5 represents the maximum value). The results were analyzed using Aiken’s V coefficient, with values ≥0.80 considered acceptable. All items exceeded this threshold (average V=0.95), although some items regarding accessibility required reformulation after the first round of evaluation. The Delphi process was concluded after 2 rounds upon reaching stability in the responses (change <5% between rounds).

Likewise, during this stage, improvements were made to the EMRs module, prioritizing compliance with the Peruvian State Technical Health Standard; a compliance schedule was developed for task management; and finally, a checklist was created comparing the requirements outlined in the NTS with the functionalities included in the EHR module to assess the level or degree of implementation of the requirements within the module; Subsequently, functional tests were conducted prior to the system going live and its subsequent use by system users, such as doctors, nurses, and professionals from other areas.

### Structural Validity and Reliability

#### Data Collection

The usability instrument was administered virtually via Google Forms, in accordance with the recommendations of the CHERRIES checklist for electronic survey reporting [29]. Participation was voluntary, and informed consent was obtained digitally prior to the survey. The survey was disseminated internally to health care professionals within the hospital network and administrative staff through corporate communication channels. No monetary incentives were provided. The survey consisted of 23 mandatory questions, and incomplete surveys were excluded. To prevent multiple submissions, controlled distribution through institutional corporate channels served as a restriction mechanism. Email addresses were not collected, nor were IP addresses logged, to maintain participant anonymity. A total of 115 complete responses were included in the final analysis. The response rate could not be calculated due to the institutional distribution methods. Data were exported directly from Google Forms to a secure database and analyzed using Jamovi software.

#### Statistical Analysis

The instrument was administered via an electronic form (Google Forms) during October-November 2024. The data were analyzed using Jamovi 2.3.28 [30]. Finally, construct validity and reliability were assessed using Cronbach α and CFA [31,32]; the latter was conducted following the standard recommendation of using between 5 and 10 participants per item or at least 100 participants [33]. In this study, we opted for 5 participants per item for CFA, given that the study population met this requirement at the facility where the case study was conducted.

### Ethical Considerations

This study was not subject to full review by the ethics committee, as it meets the requirements for exemption under applicable Peruvian national regulations. Specifically, according to Ministerial Resolution No. 233-2020-MINSA, which approves the “Ethical Considerations for Health Research Involving Human Subjects” issued by the Peruvian Ministry of Health (Ministerio de Salud [MINSA]), survey-based studies that are anonymous and noninterventional are classified as low-risk or risk-free and, therefore, do not require full approval by the ethics committee. Furthermore, the study complied with Peru’s Law No. 29733 (Personal Data Protection Law) and its Regulatory Decree D.S. No. 003-2013-JUS, which guarantees the confidentiality and anonymity of participants’ data. Likewise, prior to data collection, written institutional authorization was obtained from the American Clinic of Juliaca (January 18, 2024). Informed consent was also included directly in the web-based survey instrument. Participants were informed that their responses would be collected anonymously and used exclusively for research purposes, and that their continued participation constituted implied consent. We confirm that this study falls under category (2): it was not submitted for ethical review, as it met the exemption criteria due to its anonymous, noninterventional, and survey-based nature, in accordance with the aforementioned national policy. Participants did not receive any financial or other compensation for their participation in this study.

## Results

### Instrument Developed

Following a review of the state of the art and validation by expert judges in 2 rounds, the Spanish-language instrument based on ISO 25010 was validated, as shown in Multimedia Appendix 1 [34-41].

### Content Validity Results

This instrument was the result of an evaluation by 10 expert judges who assigned a rating on a scale of 1 to 5, where 1 represents the minimum value, and 5 represents the maximum value; these results were processed using Aiken’s V statistic to assess the content validity of the aforementioned instrument with a 95% confidence level, corresponding to 1.96.

In accordance with the previous paragraph, favorable results were obtained, with the average Aiken’s V coefficient yielding a value of 0.95, indicating that the instrument has very good content validity; these results can be verified item by item in Multimedia Appendix 2, where the mean score is 4.8 and the SD is 0.37; the lower confidence interval limit is 0.84 and the upper confidence interval limit is 0.98; these results are consequently positive for the proposed instrument, which is intended to evaluate the usability of a computer tool.

Similarly, the Aiken V results were grouped by dimension, and these also showed favorable results, with the lowest score in the accessibility dimension (an average of 0.93) and the highest score in the operability dimension (an average of 0.97), as shown in Table 1.

**Table 1.** Aiken V results by dimension.

Dimension	Recognition of suitability	Learnability	Operability	User error protection	User interface aesthetics	Accessibility	Satisfaction	General
Relevance	0.958	0.931	0.983	0.96	0.93	0.93	0.95	0.949
Relevance	0.967	0.938	0.958	0.94	0.95	0.94	0.94	0.948
Clarity	0.950	0.969	0.975	0.97	0.95	0.93	0.95	0.957
TOTAL	0.958	0.946	0.972	0.956	0.944	0.933	0.947	0.951

Additionally, the medical records module has been adapted to meet the requirements of the Peruvian State Technical Health Standard (NTS); first, a list of requirements based on the NTS was compiled; subsequently, the corresponding adjustments were made to the EHR module; this was managed through a task schedule; and finally, a checklist was created comparing the requirements implemented according to the NTS with the functionalities of the module in question; resulting in an 80% implementation of the requirements, with 20% remaining pending based on the total number of requirements; these were classified according to the following options: YES (Implemented), NO (Not Implemented), and Partial (Partially Implemented), with scores of 1, 0, and 0.5, respectively. The aforementioned results can be found in Multimedia Appendix 3.

### Psychometric Evaluation Results

The results of the pilot usability evaluation study with the developed instrument allowed for CFA, a statistical technique used to verify that the data from a model fit a predefined theoretical structure. Jamovi was used as the statistical analysis tool, yielding favorable results for the instrument in question: the CFI (Comparative Fit Index) has a value of 1, which measures model fit, and the scaled value is 0.994, indicating a perfect fit and a very good fit, respectively; likewise, the Tucker-Lewis Index has a value of 1.001 and the scaling of 0.993, which shows an excellent fit in both cases; similarly, this is the case for the Bentler-Bonett Norm-Normed Fit Index, where the model is 1.001 and the scaled value is 0.993, both indicating an excellent fit and a very robust model; on the other hand, the Relative Noncentrality Index measures relative noncentrality fit; the result for the model is 1.001 and the scaled value is 0.994, both of which also suggest an excellent fit; As for the NFI (Bentler-Bonett Normed Fit Index), which compares the model to the null model, the values are favorable, with the model at 0.997 and the scaled value at 0.980, indicating a base model with an excellent fit; Regarding the Bollen’s Relative Fit Index, the model corresponds to 0.997 and the scaled value to 0.976, similarly indicating a good fit; these results can also be observed in Multimedia Appendix 4.

**Figure 2.**
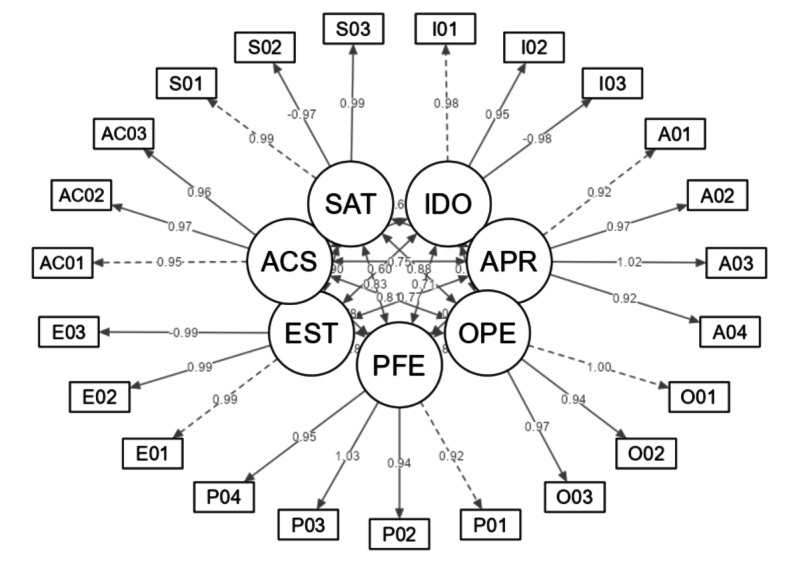
Usability evaluation model. ACS: Accesibilidad (Accessibility); APR: Aprendizabilidad (Learnability); EST: Estética (Esthetics); IDO: Idoneidad (Appropriateness); OPE: Operatividad (Operability); PFE: Protección Frente a Errores (Error Protection); SAT: Satisfacción (Satisfaction).

The results of the proposed usability measurement instrument model align with the predefined theoretical construct, as can also be seen in Figure 2.

Additionally, the instrument’s reliability was calculated using Cronbach α based on the results, yielding a value of .968, as shown in the results processed by Jamovi (Multimedia Appendix 5). This demonstrates high internal consistency and a high correlation among the items in the measurement of the construct.

Finally, the results of applying the instrument developed in accordance with ISO 25010 were obtained; for this purpose, the same principles proposed by John Brook in the development of the scales were considered to subsequently generate the results by dimension and in general. Although there are several techniques for processing SUS results [42], it was deemed appropriate to work with the rating scale technique, given that most existing instruments in other areas use the rating scale technique; therefore, the score ranges shown in Table 2 were considered to obtain the results.

**Table 2.** Scales for evaluating usability and its dimensions.

Dimension	Very low	Low	Moderate	High	Very high
Recognition of suitability (3-15)	3‐5	6‐8	9‐11	12‐13	14‐15
Learnability (4-20)	4‐7	8‐11	12‐14	15‐17	18‐20
Operability (3-15)	3‐5	6‐8	9‐11	12‐13	14‐15
User error protection (4-20)	4‐7	8‐11	12‐14	15‐17	18‐20
User Interface Esthetics (3-15)	3‐5	6‐8	9‐11	12‐13	14‐15
Accessibility (3-15)	3‐5	6‐8	9‐11	12‐13	14‐15
Satisfaction (3-15)	3‐5	6‐8	9‐11	12‐13	14‐15
Overall result					
Usability (23-115)	23‐42	43‐61	62‐79	80‐97	98‐115

Based on the above rating scale table, the following descriptive statistics were obtained regarding the results for each dimension of the Usability Factor assessment tool for computer products, as shown in Multimedia Appendix 6.

Regarding the overall usability factor score based on the developed instrument, the following descriptive statistics were obtained: mean 4.82, median 5, mode 5.00, sum 554, SD 0.388, IQR 0.00, minimum 4, and maximum 5. These results indicate a high overall usability factor score.

Referring to the frequency table of the overall result, it can be seen that 81.7% of users perceive usability to be very high, and 18.3% of users perceive it to be high, thus demonstrating that the EHRs Module used by the institution in the case study meets the usability criteria for a software product, as shown in Table 3.

However, descriptive differences were found in usability scores among professional groups, with administrative staff obtaining slightly higher scores than clinical staff, as shown in Multimedia Appendix 7. No inferential statistical comparisons were performed.

**Table 3.** Frequency of the usability factor.

Usability	Counts	Percentage of total (%)	Cumulative percentage (%)
High	21	18.3	18.3
Very high	94	81.7	100.0

## Discussion

### Main Findings

This study successfully developed and validated a usability evaluation instrument based on ISO 25010 with excellent psychometric properties (Aiken’s V=0.95, Cronbach *α*=0.968, CFI=1.000). Application of the instrument revealed that 94 of 115 users (81.7%) perceived the usability of the EHR module as very high, while the module met 28 of 35 requirements established by the Peruvian NTS (80%). Descriptive differences in usability perception were identified between clinical and administrative professionals. Highlighting three main findings: (1) Good results in psychometric properties, (2) positive perception of usability with slight differences between professional areas, and finally, partial compliance with the Peruvian NTS.

Beyond local application, this work supports the global conversation on the digital transformation of health care. The World Health Organization’s Digital Health Strategy highlights the need for contextualized evaluation frameworks to ensure that digital health systems are safe, effective, and people-centered. In this vein, usability is not merely a software feature but a factor that influences clinical performance, patient safety, and professional well-being. Poor usability of RHRs has been linked globally to increased cognitive load, documentation burden, and burnout among clinical staff. By combining the usability criteria of the ISO 25010 standard with local regulatory compliance requirements, this work proposes a framework to evaluate not only the system’s functionality but also the alignment between technological design and actual clinical workflows. However, most validated usability tools have been developed in high-income contexts and may not fully represent the sociotechnical realities of low- and middle-income countries, where infrastructure limitations, fragmented medical records, and evolving regulatory frameworks pose unique challenges. This instrument addresses this gap by operationalizing usability within a dual framework: international quality standards and national health regulations. This approach transforms usability scores into policy-driven digital health governance, making usability evaluation a tool for strengthening the health system, rather than merely a postimplementation metric.

### Comparison With Other Research

Following a systematic review, similar studies have been identified in European countries; these place significant emphasis on measuring the acceptance of hospital HIS (Hospital Information Systems) [43] through models such as the technology acceptance model and unified theory of acceptance and use of technology, where nurses are the users with the highest ratings and training is identified as one of the determining factors. On the other hand [44], a study reveals that both the technology acceptance model and unified theory of acceptance and use of technology remain valid models for predicting the acceptance of HIS by health care professionals in hospital settings. Likewise [45-47], provided qualitative and mixed methods evidence regarding the perceptions of internal users regarding HIS in European hospitals. Meanwhile, in the Latin American context, there is a scarcity of studies related to the usability or acceptance of hospital HIS; likewise [48,49], confirm that, in general, there is significantly less scientific output in digital health due to structural reasons, given that scientific output is concentrated more in high-income countries [50,51]; therefore, after an exhaustive search, only 2 studies [52,53] were identified that were closest to implementation in the Latin American context; one was implemented in Mexico and the other in Brazil; neither evaluated acceptance factors. However, a meta-analysis study [54] conducted across multiple international contexts on professional burnout related to the use of EHRs indicates that insufficient acceptance of the system has negative consequences for the well-being of health care personnel. Hence the importance of evaluating internal users’ acceptance of hospital information systems, as was done in this study; where the perceived level of the MHC’s (Módulo de Historias Clínicas [Medical Records Module]) usability is very high at 81.7% and high at 18.3%; whereas in other studies [55] relatively related to usability evaluation, results were higher than those obtained in this study, with a score of 83.1; however, a direct comparison is not possible since the calculation method in this study is based on percentages, whereas the cited study on OpenEDC evaluation uses numerical scores; the cited study involved 16 users, while our case study had a population of 115 users.

On the other hand, there is also another study [56] related to metadata management that evaluates the usability of a metadata repository called CoMetaR; 12 individuals participated in the study, in which 3 modules were evaluated, obtaining scores of 81, 81, and 72, respectively. For this, they used the SUS, which is one of the most widely used instruments for usability evaluation.

Regarding the subject of the study [57], the authors report that they conducted a systematic review in which they observed, across several primary studies, the existence of technical and social challenges in achieving quality in health information systems; according to the studies examined, various tools were used to assess software quality, including SUS, After-Scenario Questionnaire, and Nielsen’s heuristics, among others; however, only 3 out of 17 studies included the processes of ISO/IEC 25010 and ISO 9126; thus, they noted that there is no precise description of how to translate these standards into practice; that is, there is a lack of success stories, case studies, and other materials describing lessons learned regarding the application of quality standards. In line with the statements of the cited authors, this study addressed the development of a usability evaluation scale based on ISO 25010 to serve as a reference for future work related to the quality of software products.

In another systematic review [58] regarding the evaluation of usability in telemedicine tools, the authors found that many studies did not use standardized instruments, which hindered an adequate description of the usability of telemedicine tools; although it is reported that most of the studies included in the systematic review demonstrate good usability, this is not specified in percentage or numerical terms, which would allow for comparisons or the identification of improvements; on the other hand, they also note that most studies use patients as the study population for usability and very rarely internal users; even here, the situation differs in the European context or in high-income countries compared with Latin American countries such as Peru. In line with these findings and in contrast to them, this study evaluated the usability of the MHC among internal users using a validated instrument based on ISO 25010, presenting statistical data on content validity, reliability, and CFA (structural validity) to serve as a foundation for future research.

Likewise, another systematic review study [59] on usability evaluation in mobile apps for physical rehabilitation reports that there are a variety of approaches and instruments for such evaluation; that many of them lack a theoretical foundation and psychometric properties regarding usability evaluation instruments; given that they found a large number of custom-developed instruments, such as hybrid instruments or those adapted from existing ones, the latter being limited by sample size, which impairs their validity; this highlights the need for alternative approaches to increase flexibility while ensuring the psychometric properties of the instruments; on the other hand, regarding the theoretical foundation of usability, they found gaps in the connection with theoretical usability models such as the ISO standards or similar ones; in contrast to the gaps perceived and reported in systematic review research studies. This research study has sought to address the reported gaps by developing an evaluation instrument based on ISO 25010, while also incorporating its corresponding psychometric properties, although it has limitations regarding cross-cultural validity that could be addressed in future studies.

It has been observed that most systematic review studies have limitations that prevent an adequate analysis; since existing research in the literature lacks standards or guidelines to facilitate an adequate analysis of the usability of IT products, as reported by [60] in their study evaluating the usability of portable devices and complementary health applications; there, they found a variety of instruments used to assess usability, yet one that outnumbered other instruments was the SUS; despite this, only 16 of the 68 studies included in the study used any statistical analysis, and only one-third of the studies explicitly reported the measurement of usability or perceived usability; however, they had difficulty distinguishing the evaluation of the wearable device from that of the accompanying mobile app; thus highlighting the lack of standardized frameworks that allow for the proper evaluation of the usability of wearable devices and complementary apps; demonstrating the need for further research to bridge the existing gap between usability and user experience.

### Limitations

Although this study provides a robust instrument, some limitations suggest avenues for future research: the sample was limited to an urban hospital network, so validation in rural areas with less technological infrastructure is recommended. Likewise, it would be valuable to: (1) evaluate the impact of improving specific dimensions (eg, accessibility) on real clinical indicators (eg, reduction in medical errors), and (2) develop implementation guidelines that link usability results to user-centered design protocols. These lines of work could bridge the gap between evaluation and best practices in EHR systems, transforming usability from an abstract concept into a measurable driver of health care quality and patient safety in the region. Despite the limitations, the instrument provides comprehensive psychometric validation based on ISO 25010, paving the way for future research on cross-cultural validation and application in diverse contexts.

### Conclusions

This study developed and validated an instrument for evaluating the usability of information systems, which is aligned with the dimensions of ISO 25010. The results demonstrated excellent psychometric properties, with a near-perfect factor fit (CFI=1.000, Tucker-Lewis Index=1.001) and exceptional reliability (Cronbach *α*=0.968). The application of the instrument in a real-world setting revealed that 81.7% of users perceived usability as “very high,” validating not only the tool itself but also the importance of considering local regulatory frameworks in the design of digital health systems, such as Peru’s NTS. These findings represent a significant advance over generic instruments such as the SUS, by offering systematic alignment with ISO 25010, and, on the other hand, the alignment of information systems with local legislation (NTS).

The research identified certain differences in the perception of usability according to professional roles: while administrative staff reported higher satisfaction (93% “very high”), doctors and nurses gave more critical ratings (80%), particularly in dimensions such as “error protection” and “operability.” These findings align with the alignment or adaptation of information systems to the NTS, but provide specific solutions for the Peruvian context. The main practical implications include: (1) the need to redesign workflows for critical clinical tasks, (2) prioritizing ongoing training focused on usability, and (3) establishing periodic evaluations using this instrument as part of the continuous improvement processes for EHR systems. Additionally, the study revealed that 20% of the NTS requirements (primarily interoperability) were not implemented, highlighting urgent areas for technological development.

## Supplementary material

10.2196/81377 Multimedia Appendix 1 Instrument according to ISO 25010.

10.2196/81377 Multimedia Appendix 2 Content validity results by experts.

10.2196/81377 Multimedia Appendix 3 Requirements implementation checklist according to NTS (Norma Técnica de Salud (Technical Health Standard).

10.2196/81377 Multimedia Appendix 4 Results of Jamovi statistical analysis evaluation.

10.2196/81377 Multimedia Appendix 5 Cronbach alpha results.

10.2196/81377 Multimedia Appendix 6 Descriptive results of the usability dimensions.

10.2196/81377 Multimedia Appendix 7 Results of MHC usability perception by profession.

## Abbreviations

CFA: confirmatory factor analysis
CFI: Comparative Fit Index
COSMIN: Consensus-based Standards for the Selection of Health Measurement Instruments
EHR: electronic health record
EMR: electronic medical record
HCE: Historia Clínica Electrónica (Electronic Health Record)
HCM: Historia Clínica Manual (manual medical record)
HIS: Hospital Information Systems
IEC: International Electrotechnical Commission
IPRESS: Instituciones Prestadoras de Servicios de Salud (Healthcare Service Provider)
ISO: International Organization for Standardization
ISPOR: International Society for Pharmacoeconomics and Outcomes Research
IT: information technology
MHC: Módulo de Historias Clínicas (Medical Records Module)
MINSA: Ministerio de Salud (Peruvian Ministry of Health)
NFI: Normed Fit Index
NTS: Norma Técnica de Salud (Technical Health Standard)
RENHICE: Registro Nacional de Historias Clínicas Electrónicas (National Registry of Electronic Medical Records)
RHC: Medical Record Registry
SUS: System Usability Scale
