# Supplementary material for: Development and Psychometric Validation of a Usability Instrument Based on ISO 25010 for Electronic Health Record Systems in Peruvian Health Care Settings: Methodological Study
Source: JMIR Hum Factors. 2026 May 22;13:e81377. doi: 10.2196/81377 (PMC13197157; doi:10.2196/81377)
Supplement: Multimedia Appendix 1 [file humanfactors-v13-e81377-s001.docx]

**Multimedia Appendix 1.** Instrument according to ISO 25010.

| **Quality attributes ISO 25010** | **Description** | **Items in Spanish** | **Theoretical Construct** |
| --- | --- | --- | --- |
|  |  |  | **Reference** |
| **Recognition of suitability** | Product capability that allows the user to understand whether the software is suitable for their needs. | 1.- La herramienta posee las funcionalidades principales necesarias para su adecuación funcional. | Question created according to ISO/IEC 25010 [1]. |
|  |  | 2.- La herramienta permite completar las tareas de manera satisfactoria mostrando el estado o paso correspondiente. |  |
|  |  | 3.-La herramienta permite completar los procesos específicos, considerando las tareas conforme a los roles y responsabilidades de cada usuario. | Adapted according to items 3-5 [2] |
| **Learnability** | The ability of a product to enable users to learn how to use it. | 4.- Considero que la herramienta es fácil de usar. | SUS [3] |
|  |  | 5.- Es probable que la mayoría de las personas aprenderían a usar esta herramienta rápidamente. | Adapted from SUS [3] |
|  |  | 6.- La interfaz de usuario de la herramienta es bastante intuitiva. | Adapted according to SUS Positivizado [4] |
|  |  | 7.- Considero que las personas adoptarían rápidamente esta herramienta después de probarla | Question created according to Nielsen's Heuristics[5] |
| **Operability** | Product capability that allows the user to operate and control it with ease. | 8.- Considero que las funciones de la herramienta están bien integradas | SUS [3] |
|  |  | 9.- Considero que la herramienta puede utilizarse sin necesidad de apoyo por parte de personal experto. | Adapted according to SUS Positivizado [4] |
|  |  | 10.- El tiempo de respuesta ante una petición es aceptable. | Adapted according to SUS Improved Item 18. [5] |
| **User error protection** | Ability of the product to protect users from making mistakes | 11.- La herramienta cuenta con validaciones de datos de entrada, mensajes de error o alerta y confirmaciones antes de realizar acciones críticas. | Adapted from Table 4-Item 1. [7] |
|  |  | 12.- Existe mensajes de ayuda en la interfaz de usuario para realizar acciones sin errores. | Adapted from Table 4-Item 2. [7] |
|  |  | 13.- La interfaz de usuario tiene solo opciones válidas y campos con valores predeterminados. | Question created according to principle 5 [5] |
|  |  | 14.- La herramienta proporciona mensajes claros en caso de errores, facilitando su corrección. | Adapted from Table 4-Item 3. [7] |
| **User Interface Aesthetics** | Ability of the user interface to please and satisfy user interaction | 15.- El diseño de la interfaz de usuario, incluyendo el contraste de colores, íconos y tipografía, es atractivo. | Adapted according to SUS Improved Item 16. [6] |
|  |  | 16.- El diseño de la interfaz de usuario es sencillo, con pocos elementos innecesarios y bien organizado. | Question created according to principle 8 [5] |
|  |  | 17.- El diseño de la interfaz de usuario genera tranquilidad y confianza. | Question created according to [8] |
| **Accessibility** | Product capability that allows it to be used by users with certain characteristics and disabilities. | 18- El diseño responsivo de la herramienta asegura la adaptabilidad de la interfaz de usuario a cualquier dispositivo y o tamaño de pantalla. | Question created according to the definition in ISO 25010, sub-feature Accessibility. [1] |
|  |  | 19.- La herramienta ofrece soporte para discapacidades visuales, auditivas, motoras y/o cognitivas. |  |
|  |  | 20.- La herramienta valida que el contenido de la interfaz se vea y opere correctamente con lectores de pantalla como JAWS, NVDA o VoiceOver. |  |
| **Satisfaction** | The product's ability to generate satisfaction in use. | 21.- Estoy satisfecho al usar esta herramienta | Adapted according to SUS Positivizado [4] |
|  |  | 22.- Considero que la herramienta muestra coherencia y consistencia notable según su propósito | Adapted according to Item 6 SUS [3] |
|  |  | 23.- Me gustaría usar esta herramienta frecuentemente. | SUS [3] |

References

1. International Organization for Standardization (ISO). ISO/IEC 25010:2011(en), systems and software engineering — systems and software quality requirements and evaluation (SQuaRE) — system and software quality models. 2011. Accessed January 16, 2024. https://www.iso.org/obp/ui/#iso:std:iso-iec:25010:ed-1:v1:en

2. Lewis JR. IBM computer usability satisfaction questionnaires: psychometric evaluation and instructions for use. Int J Hum Comput Interact. 1995;7(1):57-78. doi:10.1080/10447319509526110

3. Sevilla-Gonzalez MDR, Moreno Loaeza L, Lazaro-Carrera LS, et al. Spanish version of the system usability scale for the assessment of electronic tools: development and validation. JMIR Hum Factors. 2020;7(4):e21161. doi:10.2196/21161

4. Lewis JR. The system usability scale: past, present, and future. Int J Hum Comput Interact. 2018;34(7):577-590. doi:10.1080/10447318.2018.1455307

5. Nielsen J. 10 usability heuristics for user interface design. 2020. Accessed January 16, 2024. https://www.nngroup.com/articles/ten-usability-heuristics/

6. Thamilarasan Y, Raja Ikram RR, Osman M, Salahuddin L, Wan Bujeri WY, Kanchymalay K. Enhanced system usability scale using the software quality standard approach. Engineering, Technology and Applied Science Research. 2023;13(5):11779-11784. doi:10.48084/etasr.5971

7. Chanchí EG, Gómez Álvarez MC, Yesid Campo WM. Propuesta de una herramienta de inspección según los atributos de usabilidad de Nielsen. 2023. Accessed January 16, 2024. https://www.proquest.com/openview/b15b1d1ae0fd63a1471d3fbd3faf6cd9/1?pq-origsite=gscholar&cbl=1006393

8. Zen M, Burny N, Vanderdonckt J. A quality model-based approach for measuring user interface aesthetics with Grace. Proc ACM Hum Comput Interact. 2023;7(EICS):172. doi:10.1145/3593224
