# Supplementary material for: Development and Psychometric Validation of a Usability Instrument Based on ISO 25010 for Electronic Health Record Systems in Peruvian Health Care Settings: Methodological Study
Source: JMIR Hum Factors. 2026 May 22;13:e81377. doi: 10.2196/81377 (PMC13197157; doi:10.2196/81377)
Supplement: Multimedia Appendix 2 [file humanfactors-v13-e81377-s002.pdf]

Multimedia Appendix 2. Content validity results by experts.

Items	Criterion	J1	J2	J3	J4	J5	J6	J7	J8	J9	J10	Median	SD	Aiken's V	IC. Lower	IC. Higher
**Item1**	**Relevance**	5	4	5	5	5	5	5	5	5	4	4.80	0.42	0.95	0.83	0.99
	**Pertinence**	5	5	5	5	5	5	5	5	5	5	5.00	0.00	1.00	0.91	1.00
	**Clarity**	5	5	5	5	5	5	5	5	5	4	4.90	0.32	0.98	0.87	1.00
**Item2**	**Relevance**	5	5	5	5	5	5	5	5	5	4	4.90	0.32	0.98	0.87	1.00
	**Pertinence**	5	5	5	5	5	5	5	5	5	5	5.00	0.00	1.00	0.91	1.00
	**Clarity**	5	5	5	5	5	5	5	5	5	4	4.90	0.32	0.98	0.87	1.00
**Item3**	**Relevance**	5	4	5	5	5	5	5	5	5	4	4.80	0.42	0.95	0.83	0.99
	**Pertinence**	5	4	5	5	5	5	5	4	4	4	4.60	0.52	0.90	0.77	0.96
	**Clarity**	4	4	5	5	5	5	5	5	4	4	4.60	0.52	0.90	0.77	0.96
**Item4**	**Relevance**	5	4	5	5	5	5	5	5	5	4	4.80	0.42	0.95	0.83	0.99
	**Pertinence**	5	4	5	5	5	5	5	5	4	4	4.70	0.48	0.93	0.80	0.97
	**Clarity**	5	4	5	5	5	5	5	5	5	4	4.80	0.42	0.95	0.83	0.99
**Item5**	**Relevance**	5	4	5	5	5	5	5	5	4	4	4.70	0.48	0.93	0.80	0.97
	**Pertinence**	5	4	5	5	5	5	5	4	4	4	4.60	0.52	0.90	0.77	0.96
	**Clarity**	5	5	5	5	5	5	5	5	5	4	4.90	0.32	0.98	0.87	1.00
**Item6**	**Relevance**	5	4	5	5	5	5	5	4	4	4	4.60	0.52	0.90	0.77	0.96
	**Pertinence**	5	4	5	5	5	5	5	5	4	4	4.70	0.48	0.93	0.80	0.97
	**Clarity**	5	5	5	5	5	5	5	5	5	4	4.90	0.32	0.98	0.87	1.00
**Item7**	**Relevance**	5	4	5	5	5	5	5	5	5	4	4.80	0.42	0.95	0.83	0.99
	**Pertinence**	5	5	5	5	5	5	5	5	5	5	5.00	0.00	1.00	0.91	1.00
	**Clarity**	5	5	5	5	5	5	5	5	5	4	4.90	0.32	0.98	0.87	1.00
**Item8**	**Relevance**	5	4	5	5	5	5	5	5	5	4	4.80	0.42	0.95	0.83	0.99
	**Pertinence**	5	4	5	5	5	5	5	5	4	4	4.70	0.48	0.93	0.80	0.97
	**Clarity**	5	5	5	5	5	5	5	5	5	4	4.90	0.32	0.98	0.87	1.00
**Item9**	**Relevance**	5	5	5	5	5	5	5	5	5	5	5.00	0.00	1.00	0.91	1.00
	**Pertinence**	5	5	5	5	5	5	5	4	5	5	4.90	0.32	0.98	0.87	1.00
	**Clarity**	5	5	5	5	5	5	5	5	5	4	4.90	0.32	0.98	0.87	1.00
**Item10**	**Relevance**	5	5	5	5	5	5	5	5	5	5	5.00	0.00	1.00	0.91	1.00
	**Pertinence**	5	5	5	5	5	5	5	5	4	5	4.90	0.32	0.98	0.87	1.00
	**Clarity**	5	5	5	5	5	5	5	5	5	4	4.90	0.32	0.98	0.87	1.00
**Item11**	**Relevance**	5	4	5	5	5	5	5	5	5	4	4.80	0.42	0.95	0.83	0.99
	**Pertinence**	5	4	5	5	5	5	5	5	4	4	4.70	0.48	0.93	0.80	0.97
	**Clarity**	5	4	5	5	5	5	5	5	5	4	4.80	0.42	0.95	0.83	0.99
**Item12**	**Relevance**	5	5	5	5	5	5	5	5	5	5	5.00	0.00	1.00	0.91	1.00
	**Pertinence**	5	5	5	5	5	5	5	5	4	5	4.90	0.32	0.98	0.87	1.00
	**Clarity**	5	5	5	5	5	5	5	5	5	4	4.90	0.32	0.98	0.87	1.00
**Item13**	**Relevance**	5	5	5	5	5	5	5	4	5	5	4.90	0.32	0.98	0.87	1.00
	**Pertinence**	5	4	5	5	5	5	5	5	4	4	4.70	0.48	0.93	0.80	0.97
	**Clarity**	5	5	5	5	5	5	5	5	5	4	4.90	0.32	0.98	0.87	1.00
**Item14**	**Relevance**	5	4	5	5	5	5	5	5	4	4	4.70	0.48	0.93	0.80	0.97
	**Pertinence**	5	4	5	5	5	5	5	5	4	4	4.70	0.48	0.93	0.80	0.97
	**Clarity**	5	5	5	5	5	5	5	5	5	4	4.90	0.32	0.98	0.87	1.00
**Item15**	**Relevance**	5	5	5	5	5	5	5	5	4	4	4.80	0.42	0.95	0.83	0.99
	**Pertinence**	5	4	5	5	5	5	5	5	4	4	4.70	0.48	0.93	0.80	0.97
	**Clarity**	5	5	5	5	5	5	5	5	5	4	4.90	0.32	0.98	0.87	1.00
**Item16**	**Relevance**	5	4	5	5	5	5	5	5	4	4	4.70	0.48	0.93	0.80	0.97
	**Pertinence**	5	5	5	5	5	5	5	5	5	5	5.00	0.00	1.00	0.91	1.00
	**Clarity**	5	5	5	5	5	5	5	4	5	4	4.80	0.42	0.95	0.83	0.99
**Item17**	**Relevance**	5	4	5	5	5	5	5	5	4	4	4.70	0.48	0.93	0.80	0.97
	**Pertinence**	5	4	5	5	5	5	5	5	4	4	4.70	0.48	0.93	0.80	0.97
	**Clarity**	5	4	5	5	5	5	5	5	4	4	4.70	0.48	0.93	0.80	0.97
**Item18**	**Relevance**	5	4	5	5	5	5	5	5	4	4	4.70	0.48	0.93	0.80	0.97
	**Pertinence**	5	4	5	5	5	5	5	5	5	4	4.80	0.42	0.95	0.83	0.99
	**Clarity**	5	4	5	5	5	5	5	5	4	4	4.70	0.48	0.93	0.80	0.97
**Item19**	**Relevance**	5	4	5	5	5	5	5	5	4	4	4.70	0.48	0.93	0.80	0.97
	**Pertinence**	5	4	5	5	5	5	5	5	5	4	4.80	0.42	0.95	0.83	0.99
	**Clarity**	5	5	5	5	5	5	5	5	5	4	4.90	0.32	0.98	0.87	1.00
**Item20**	**Relevance**	5	4	5	5	5	5	5	5	4	4	4.70	0.48	0.93	0.80	0.97
	**Pertinence**	5	4	5	5	5	5	5	5	4	4	4.70	0.48	0.93	0.80	0.97
	**Clarity**	5	4	5	5	5	5	5	4	4	4	4.60	0.52	0.90	0.77	0.96
**Item21**	**Relevance**	5	5	5	5	5	5	5	4	4	4	4.70	0.48	0.93	0.80	0.97
	**Pertinence**	5	5	5	5	5	5	5	5	4	5	4.90	0.32	0.98	0.87	1.00
	**Clarity**	5	5	5	5	5	5	5	5	4	4	4.80	0.42	0.95	0.83	0.99
**Item22**	**Relevance**	5	5	5	5	5	5	5	5	5	4	4.90	0.32	0.98	0.87	1.00
	**Pertinence**	5	4	5	5	5	5	5	5	4	4	4.70	0.48	0.93	0.80	0.97
	**Clarity**	5	4	5	5	5	5	5	5	5	4	4.80	0.42	0.95	0.83	0.99
**Item23**	**Relevance**	5	4	5	5	5	5	5	5	5	4	4.80	0.42	0.95	0.83	0.99
	**Pertinence**	5	4	5	5	5	5	5	5	4	4	4.70	0.48	0.93	0.80	0.97
	**Clarity**	5	4	5	5	5	5	5	5	5	4	4.80	0.42	0.95	0.83	0.99
