# Supplementary material for: Development and Psychometric Validation of a Usability Instrument Based on ISO 25010 for Electronic Health Record Systems in Peruvian Health Care Settings: Methodological Study
Source: JMIR Hum Factors. 2026 May 22;13:e81377. doi: 10.2196/81377 (PMC13197157; doi:10.2196/81377)
Supplement: Multimedia Appendix 3 [file humanfactors-v13-e81377-s003.pdf]

Multimedia Appendix 3. Requirements implementation checklist according to NTS.

Sub-Module	Requirement: Electronic Medical Record Module in accordance with NTS	Does your medical record module currently include this feature?
Clinical Record Management	1.- Registration of medical records considering basic data, care data, and complementary information	YES
	2.- Organization of medical records in chronological order from most recent to oldest	YES
	3.- Record of clinical care using standardised formats (ICD-10 for diagnosis) according to age group (child, adolescent, young adult, adult and older adult).	YES
	4.- Record stories according to area of care, such as outpatient, emergency, and/or hospitalisation.	YES
	5.- Management of special forms such as references and/or counter-references or inter-consultations.	Partial
	6.- Record of laboratory tests or images.	YES
	7.- Correction of medical records when necessary, following a defined process, without deleting the previous medical record.	YES
Security and Confidentiality Management	8.- Management of roles and access privileges to electronic medical records.	YES
	9.- Authentication with digital signatures or unique credentials.	YES
	10.- Encryption of sensitive data.	YES
	11.- Informed consent management and data protection.	YES
	12.- Management and control of open sessions, allowing monitoring.	YES
Electronic and Digital Signature Management	13.- Management of digital and/or electronic signatures of medical personnel.	YES
	14.- Validation through the use of a digital signature by the treating physician to endorse diagnoses, prescriptions, epicrisis, medical reports, and informed consent.	YES
	15.- Use of electronic signatures for common medical records such as laboratory results, prescriptions, and reports.	YES
	16.- Validation of digital document certificates.	NO
Clinical History File Management	17.- Electronic medical record lifecycle management in accordance with current regulations.	Partial
	18.- Classification of medical records according to each case: active, passive, and special.	NO
	19.- Deletion of medical records, change of status.	NO
Report Management	20.- Search for medical records	YES
	21.- Reports of medical records by status type, such as active, passive, special, and deleted.	YES
	22.- Key statistical data for the management of IPRESS	YES
	23.- Report on user access and actions.	YES
	24.- Reports on administrative activities related to file management.	YES
	25.- Export reports in PDF, Excel, or other formats.	YES
	26.- Clinical Report Management.	YES
Interoperability management	27.- Interoperability with RENHICE or other centralised registries.	YES
	28.- HCE consultation with RENHICE	NO
	29.- Connection to other systems such as medical insurance (SIS, SOAT), billing systems, laboratories and/or pharmacies.	NO
	30.- Interoperability between health entities.	NO

Audit Management	31.- Record of executed DMLs, including basic data such as who executed it, date, time, and action.	YES
	32.- Report on actions taken for internal or external inspections.	YES
	33.- Traceability of who signed, when, and from which device.	YES
	34.- Access to review and/or verification of medical records.	YES
	35.- Medical record access history.	YES
