# Supplementary figures and images for: Development and Psychometric Validation of a Usability Instrument Based on ISO 25010 for Electronic Health Record Systems in Peruvian Health Care Settings: Methodological Study
Source: JMIR Hum Factors. 2026 May 22;13:e81377. doi: 10.2196/81377 (PMC13197157; doi:10.2196/81377)

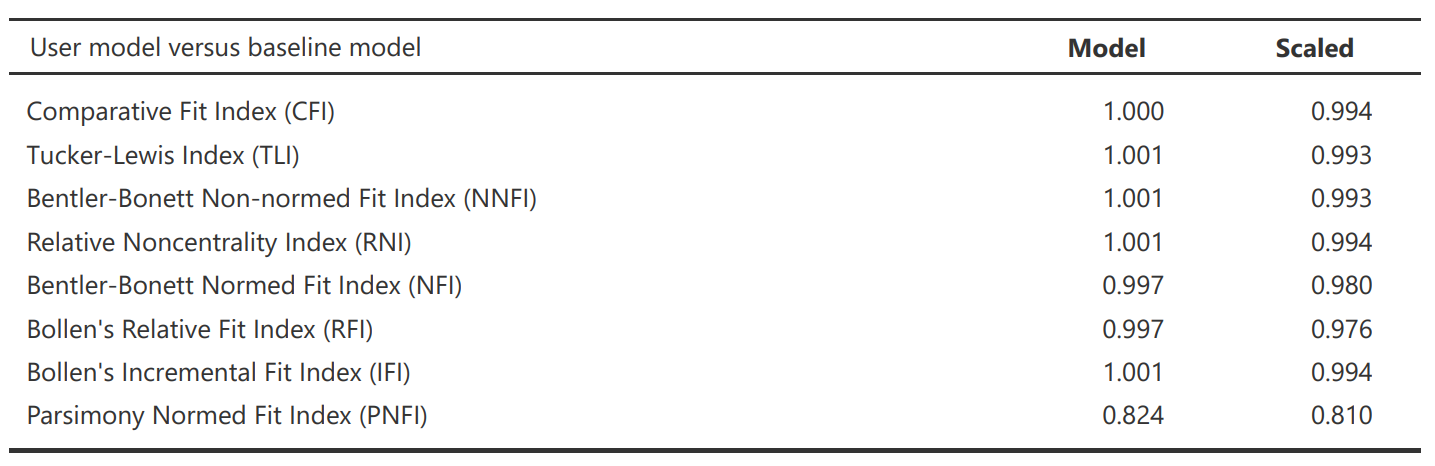

Supplement: Multimedia Appendix 4 [file humanfactors-v13-e81377-s004.png]

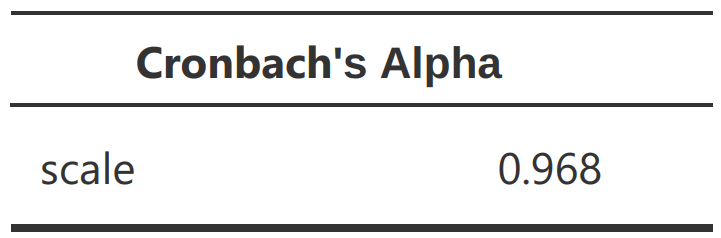

Supplement: Multimedia Appendix 5 [file humanfactors-v13-e81377-s005.png]

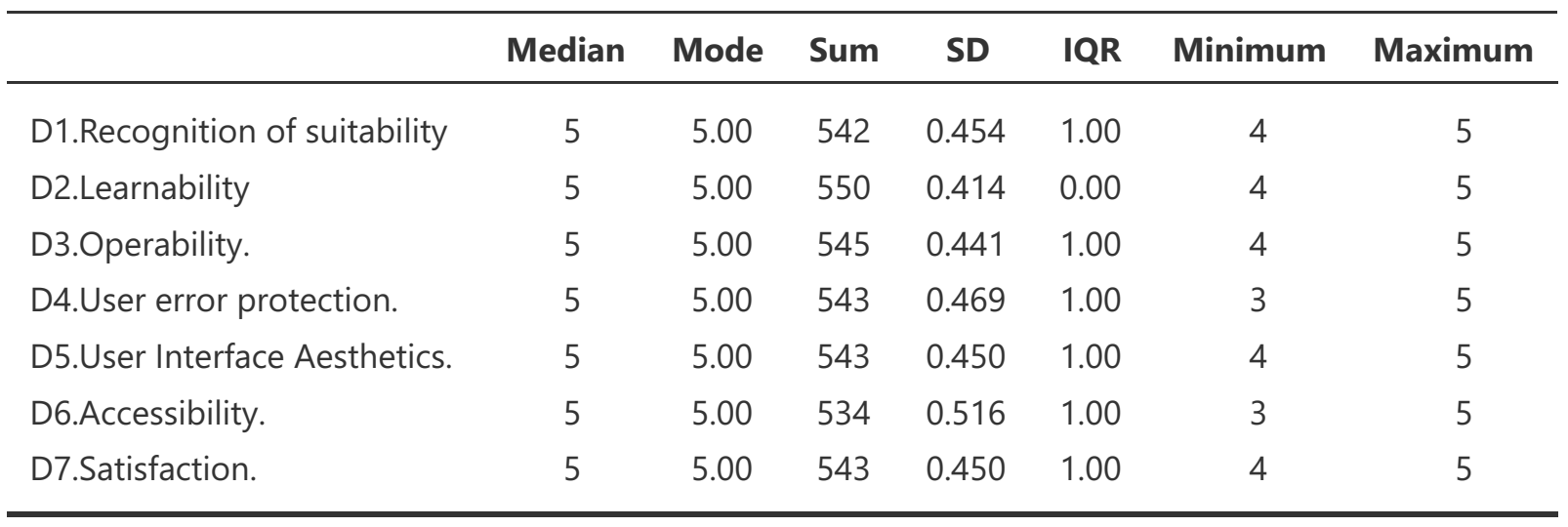

Supplement: Multimedia Appendix 6 [file humanfactors-v13-e81377-s006.png]

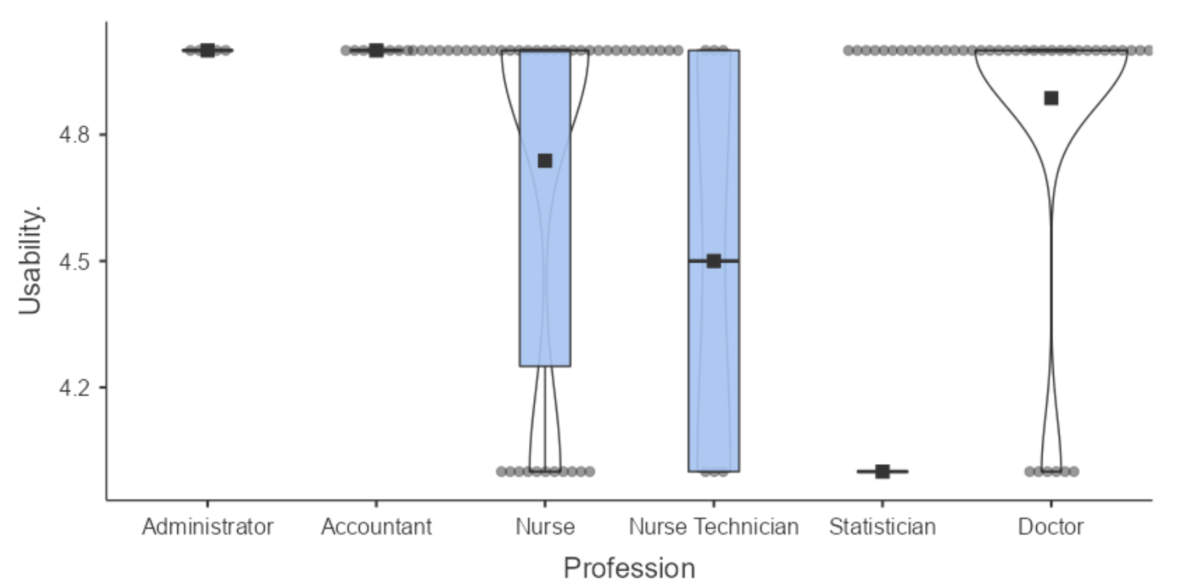

Supplement: Multimedia Appendix 7 [file humanfactors-v13-e81377-s007.png]
